# Predictors of Major Depressive Disorder following Intensive Care of Chronically Critically Ill Patients

**DOI:** 10.1155/2018/1586736

**Published:** 2018-08-01

**Authors:** Gloria-Beatrice Wintermann, Jenny Rosendahl, Kerstin Weidner, Bernhard Strauß, Katja Petrowski

**Affiliations:** ^1^Department of Psychotherapy and Psychosomatic Medicine, Medizinische Fakultät Carl Gustav Carus, Technische Universität Dresden, Dresden, Germany; ^2^Center for Sepsis Control and Care, Jena University Hospital, Friedrich-Schiller University, Jena, Germany; ^3^Institute of Psychosocial Medicine and Psychotherapy, Jena University Hospital, Friedrich-Schiller University, Jena, Germany; ^4^Institute of Medical Psychology and Medical Sociology, Clinic and Polyclinic for Psychosomatic Medicine and Psychotherapy, University Medical Center of the Johannes Gutenberg University, Mainz, Germany

## Abstract

**Objective:**

Major depressive disorder (MDD) is a common condition following treatment in the Intensive Care Unit (ICU). Long-term data on MDD in chronically critically ill (CCI) patients are scarce. Hence, the primary aim of the present study was to investigate the frequency and predictors of MDD after intensive care of CCI patients.

**Materials and Methods:**

In a prospective cohort study, patients with long-term mechanical ventilation requirements (*n*=131) were assessed with respect to a diagnosis of MDD, using the Structured Clinical Interview for DSM-IV, three and six months after the transfer from acute ICU to post-acute ICU. Sociodemographic, psychological, and clinical risk factors with *p* values ≤ 0.1 were identified in a univariate logistic regression analysis and entered in a multivariable logistic regression model. A mediator analysis was run using the bootstrapping method, testing the mediating effect of perceived helplessness during the ICU stay, between the recalled traumatic experience from the ICU and a post-ICU MDD.

**Results:**

17.6% (*n*=23) of the patients showed a full- or subsyndromal MDD. Perceived helplessness, recalled experiences of a traumatic event from the ICU, symptoms of acute stress disorder, and the diagnosis of posttraumatic stress disorder (PTSD) after ICU could be identified as significant predictors of MDD. In a mediator analysis, perceived helplessness could be proved as a mediator.

**Conclusions:**

Every fifth CCI patient suffers from MDD up to six months after being discharged from ICU. Particularly, perceived helplessness during the ICU stay seems to mainly affect the long-term evolvement of MDD. CCI patients with symptoms of acute stress disorder/PTSD should also be screened for MDD.

## 1. Background

An acute critical illness like multiple trauma, sepsis, or acute respiratory failure necessitates treatment in an Intensive Care Unit (ICU). A certain group of patients with serious comorbidities and complications need prolonged invasive ventilation, entailing an increased risk for the development of the chronic critical illness syndrome. Core feature of chronic critical illness (CCI) is a ventilator dependence of at least three weeks [1]. Additionally, further devastating clinical conditions are common, among them: neuromuscular disorders (e.g., critical illness polyneuropathy (CIP) and critical illness myopathy (CIM)), endocrinopathy, malnutrition, impaired anabolism, anasarca, and protracted delirium. CCI leads to an increased one-year mortality of about 58% and increases the risk for long-term institutional care [2, 3]. Prolonged life support with enduring dependence on mechanical ventilation set these patients at a state of debilitating physical and emotional well-being. This has been referred to as post-intensive care syndrome (PICS) [4–6]. Besides anxiety disorders and posttraumatic stress disorder (PTSD), major depressive disorder (MDD) is a common psychological long-term sequela following intensive care [7, 8].

While PTSD has been profoundly investigated following ICU [9–11] (see systematic reviews: [8, 12, 13]), the frequency and predictors of MDD have been rarely studied. In particular, data on MDD in chronically critically ill patients following long-term mechanical ventilation are scarce. Clinically relevant symptoms of depression are common in patients following critical illness with a median point prevalence of 28% [7, 14–17]. In order to ascertain a diagnosis of clinically relevant MDD, existing studies mostly used self-report measurements instead of structured clinical expert interviews [16, 17]. Furthermore, a long time frame of up to seven years following ICU was applied (e.g., [16]). Because of the heterogeneity of the applied assessment methods and time frames, estimations for MDD frequencies are supposed to be imprecise.

With respect to the risk factors, for example, duration of mechanical ventilation/ICU stay/sedation, severity of medical illness, age, and gender, no consistent association with depressive symptoms could be shown in the general ICU population [14, 16]. However, duration of delirium, early post-ICU depression, physical and cognitive impairments during post-ICU rehabilitation, as well as socioeconomic status, could be identified [14, 16–18]. Although former studies assessed peri-ICU experiences as possible risk factors for post-ICU mental disorders [12, 17, 19, 20], it is not known whether perceived helplessness in ICU and symptoms of acute stress disorder are significant risk factors for the development of a MDD in CCI patients. Early assessment of risk factors of MDD following ICU treatment is important since depressive symptoms could lead to limitations in their ability to actively participate in rehabilitation [15].

Hence, the primary aim of the present study was to investigate the frequency of MDD, using a Structured Clinical Interview and, secondly, to investigate risk factors of MDD in CCI patients. We hypothesize that symptoms of acute stress disorder are significant risk factors for the development of a MDD. Moreover, perceived helplessness during the ICU stay is assumed to mediate the impact of the recalled experience of a traumatic event from the ICU treatment on the occurrence of post-ICU MDD.

## 2. Materials and Methods

### 2.1. Setting and Procedure

The present prospective uncontrolled observational study was registered at the German Clinical Trials Register (no. DRKS00003386) and approved by the local Ethics Committee of the Friedrich-Schiller University, Jena, Germany (no. 3278-10/11). All patients gave written informed consent.

### 2.2. Participants and Sample Size

All patients meeting the following mentioned criteria were consecutively enrolled at the Bavaria Clinic Kreischa, a rehabilitation hospital with post-acute ICUs for ventilator weaning. The criteria include: principal diagnosis of a critical illness polyneuropathy (CIP; ICD: G62.80) or critical illness myopathy (CIM; ICD: G72.80), with or without sepsis, between 18 and 75 years, a minimum stay of six days in ICU, sufficient German language skills, informed consent, and a negative evaluation of the delirium assessment, confusion assessment method for the Intensive Care Unit (CAM-ICU) [21, 22]. Cognitively or sensory impaired patients (e.g., positive CAM-ICU and deaf-muteness) and patients with stupor, with insufficient German language skills, and with rapidly terminal disease states or infaust prognosis (e.g., malignancy) were excluded from the present study.

CCI patients were interviewed at three different times by trained research assistants. The first time was face-to-face at the bedside within four weeks (*t*1) after transfer from the acute ICU. Follow-up data were assessed via telephone interview three (*t*2) and six (*t*3) months after the transfer from acute ICU to post-acute ICU. Data on the course and predictors of PTSD in CCI patients following ICU have already been published elsewhere (for details, see [10, 13]).

### 2.3. Measures

The current diagnosis of MDD was assessed using the Structured Clinical Interview for the Diagnostic and Statistical Manual of Mental Disorders (DSM-IV) (SCID I) ([23, 24]) at *t*2 and *t*3. Additionally, either at *t*2 or at *t*3, patients were examined with regard to a lifetime history of MDD. A trained clinical psychologist with a minimum experience of five years applied the SCID I via a telephone interview. Retest reliability for the SCID I can be evaluated as good (kappa score range: 0.61–0.83) [25].

According to the DSM-IV, MDD was diagnosed, if at least five of the following symptoms have been present during the same 2-week period, nearly every day and the majority of the day: depressed mood (1), decreased interest, or pleasure in daily activities (2) (cluster A), significant weight change or change in appetite, sleep disturbance, disturbance in psychomotor activity, fatigue or loss of energy, feelings of guilt or worthlessness, diminished concentration or indecisiveness, and suicidality (cluster B). The depressive symptoms should cause clinically significant suffering in social and functional status. Furthermore, the symptoms should not be attributable to bereavement, substance use, a medical condition, or other psychiatric disorders. All patients with a full-syndromal MDD had to show at least one symptom from cluster A and a minimum of four symptoms from cluster B. For a subsyndromal manifestation of MDD, patients had to fulfill cluster A but failed to reach the minimum number of symptoms in cluster B. Alternatively, patients had to meet all symptom clusters but without reporting clinically significant distress in important areas of functioning.

Socioeconomic characteristics (e.g., age, family status, and education), and medical history (e.g., history of harmful alcohol consumption/anxiety or affective disorders, diagnosis of sepsis, number of sepsis episodes, site of infection, length of mechanical ventilation, length of ICU stay, and medical comorbidities), were assessed at *t*1 from the patient record forms. Functional status was assessed by a trained study nurse using the Barthel index. The Barthel index measures performance in activities of daily living in eleven domains (e.g., fecal incontinence, urinary incontinence, and help with grooming/toilet use) with values ranging between 0 and 100. A higher value is associated with a higher degree of independence from caregivers. Additionally, the early rehabilitation Barthel index was assessed considering the following seven domains: intensive care supervision, tracheostomy tube management and supervision, intermittent or continuous mechanical ventilation, confusion, behavioral disturbances, severe impairment of communication, and dysphagia. A minimum value of −325 and a maximum value of 0 can be reached [26]. Both Barthel scales are summed up, yielding scores between −325 and 100. Inter-rater reliability is very high (*r* = 0.95). Test-retest reliability is also good (*r* = 0.89) [27].

Symptoms of acute stress disorder were measured using the German version of the Acute Stress Disorder Scale (ASDS) [28] at *t*1. This scale consists of 19 items representing symptoms of reexperiencing, avoidance, arousal, and dissociation. Patients rated the extent of symptoms on a 5-point Likert scale (1 = not at all; 5 = very much). All item scores are summed up to a total score (range: 19–95). In the present study, Cronbach's *α* was 0.96. Furthermore, perceived helplessness and fear of dying in ICU were assessed using numeric rating scales (1 = not at all; 10 = completely agree).

A diagnosis of PTSD was ascertained with the SCID I via telephone contact at *t*2 and *t*3 by a trained clinical psychologist. A detailed description for the definition of a sub- or full-syndromal manifestation of PTSD can be found elsewhere (e.g., [29, 30]). The number of recalled traumatic memories from the ICU (nightmares, severe anxiety/panic, severe pain, and troubles to breathe/feelings of suffocation) was assessed at *t*2 and *t*3 according to the questionnaire by Stoll et al. [31]. Scores are summed up (range: 0–4). In the present study, Cronbach's *α* for the number of traumatic memories from ICU was 0.53.

The perceived social support by family members, friends, and significant others, was assessed using the Multidimensional Scale of Perceived Social Support (MSPSS) [32] at *t*2 and *t*3. It consists of 12 items, which are rated on a 7-point Likert scale (1 = definitely no; 7 = definitely yes). Scores of all items are summed up to a total score (range: 12–84). Cronbach's *α* was 0.89 in the present study.

Quality of life was measured using the questionnaire Euro-Quality of Life (EQ-5D-3L) [33] at *t*2 and *t*3. The EQ-5D-3L measures the health-related quality of life on five dimensions (mobility, self-care, usual activities, pain/discomfort, and anxiety/depression), which are evaluated within three severity levels (no problems, some or moderate problems, and extreme problems or unable). A single one-dimensional index value is generated based on a simple sum score according to Hinz et al. [34]. In the present study, Cronbach's á for health-related quality of life was 0.75.

### 2.4. Statistical Methods

We ascertained normal distribution by the Kolmogorov–Smirnov test, in the case of normally distributed data, arithmetic means and standard deviations, otherwise data medians and interquartile ranges, are reported. For categorical variables, absolute and relative frequencies are presented. We used Mann–Whitney *U* test or *t*-test to compare medians or means of outcome variables between followed-up and dropped-out patients. The *χ* ^2^-test or Fisher's exact test is reported, in case of nominal outcome data. For the calculation of the primary outcome, a diagnosis of MDD, we included both full- and subsyndromal cases. The frequencies of MDD at *t*2 and *t*3 were compared using the McNemar test. In order to assess the association between a diagnosis of MDD and the health-related quality of life, the point-biserial correlation coefficient was calculated. We took the correlation between a diagnosis of MDD and PTSD, applying the contingency coefficient. Univariate logistic regression analyses were run with MDD as dichotomous outcome variable, and sociodemographic, psychological, and clinical predictor variables. Odds ratios (ORs) and 95% confidence intervals were generated. Factors with a *p* value ≤ 0.1 [9, 35] were entered in a multivariable logistic regression model with forward Wald as the variable selection method. Finally, we applied mediation analysis using the bootstrapping method with bias-corrected confidence estimates [36]. The 95% confidence interval of the indirect effect was obtained with 5000 bootstrap resamples. Significance of the mediator effect was calculated using the Sobel test [37]. A significance level of *α* = 0.05 (two-sided) was chosen. All analyses were performed using SPSS 23.0.0.0 (SPSS Inc., Chicago, IL, USA).

## 3. Results

### 3.1. Descriptive Data and Dropout Analysis

**Figure 1:**
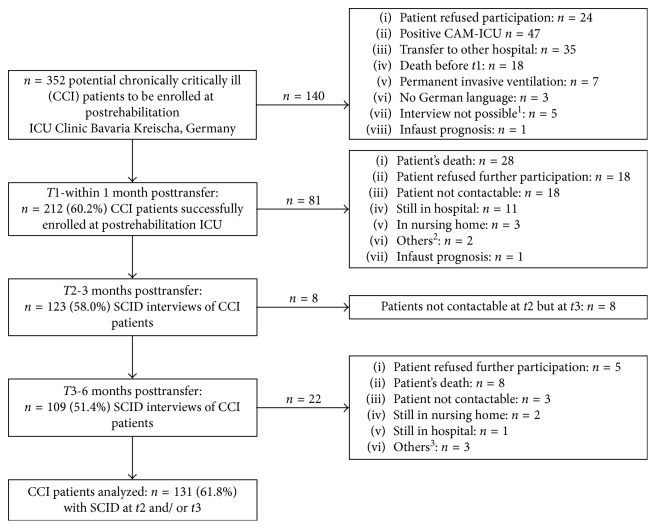
Flow diagram of the chronically critically ill patients followed up and dropped out. *N*=352 patients were recognized as potentially to be enrolled CCI patients. Of the *n*=212 successfully interviewed patients at *t*1, *n*=131 (61.8%) patients could be assessed again at *t*2 (three months after the transfer from acute ICU) and/or *t*3 (six months after the transfer from acute ICU). CAM-ICU: Confusion Assessment Method for the Intensive Care Unit. SCID: Structured Clinical Interview for DSM-IV. ^1^In *n*=2 patients, communication was not possible (only headshaking and movement of lips); in *n*=1 patient, SCID had to be interrupted because the patient was too weak; *n*=1 patient was deaf-mute; and *n*=1 was patient mentally disabled. ^2^In *n*=1 patient, communication was not possible because of patient's amblyacousia, and *n*=1 patient had permanent invasive ventilation. ^3^In *n*=1 patient, communication was not possible because of patient's amblyacousia; *n*=1 patient had permanent invasive ventilation; and in *n*=1 patient, communication was not possible because of stroke.

Of the *N*=352 potentially eligible CCI patients, *n*=131 (61.8%) patients could be followed up to six months after the transfer from acute ICU (Figure 1). Patients who were followed up had a median age of 61.2 years (range 25.6–70.8). Most patients were male (71.8%), were married or cohabitated (71.1%), and were educated 10 years or more (68.5%) (Table 1). Two-thirds of the patients had a diagnosis of sepsis (66.3%; Table 1) and were treated for acute respiratory insufficiency (Table S1, Supplementary Materials). CCI patients who dropped out after *t*1 (*n*=81; 38.2%), stayed significantly longer in ICU, were more often educated less than 10 years, and showed significantly lower Barthel indices, both at discharge from post-acute ICU and from rehabilitation hospital (Table 1). In addition, they had more often pneumonia, chronic kidney disease, and neurological disorders, and presented with a significantly higher number of secondary diagnoses (Table S1, Supplementary Materials).

### 3.2. Frequency of MDD and Its Impact on Health-Related Quality of Life and Comorbidity with PTSD up to Six Months after the Transfer from Acute ICU

Of the *N*=131 followed-up CCI patients, *n*=23 (17.6%) patients showed a full-syndromal or subsyndromal manifestation of a MDD (Table 2). At *t*2/*t*3, *n*=6 (4.9%)/*n*=6 (5.5%) of the CCI patients were diagnosed with full-syndromal MDD. The frequencies of MDD did not significantly differ between *t*2 and *t*3 (McNemar test: *p*=0.629).

A diagnosis of MDD was significantly negatively correlated with health-related quality of life (point-biserial correlation = −0.284; *p*=0.001). Compared to 13.0% of the CCI patients without MDD, 52.2% of the CCI patients with MDD also showed a full- or subsyndromal manifestation of PTSD according to the SCID I within up to six months after the transfer from acute ICU to post-acute ICU (contingency coefficient = 0.350; *p* < 0.001).

### 3.3. Predictors of MDD

In a univariate logistic regression analysis, perceived helplessness in ICU (OR = 1.88; 95% CI 1.17–3.02; *p*=0.009), symptoms of acute stress disorder (OR = 1.62; 95% CI 1.10–2.37; *p*=0.015), the recalled experience of a traumatic event from the ICU stay (OR = 2.69; 95% CI 1.05–6.90; *p*=0.039), and a diagnosis of PTSD (OR = 7.25; 95% CI 2.69–19.55; *p* < 0.001) could be identified as significant predictors of the diagnosis of MDD up to six months after the transfer to post-acute ICU (Table S2, Supplementary Materials). However, the diagnosis of acute stress disorder according to the SCID I failed to reach significance (*p*=0.051; Table S2).

The multivariable logistic regression analysis confirmed perceived helplessness in ICU (OR = 1.79; 95% CI 1.08–2.97; *p*=0.024) and a diagnosis of PTSD in the follow-up (OR = 7.41; 95% CI 2.63–20.94; *p* < 0.001) as significant predictors (Table 3). Family status, symptoms of acute stress disorder, and the recalled experience of a traumatic event from the ICU could not be proven. The model explained 25.2% of the data variance (Nagelkerke's *R* ^2^ = 0.252).

### 3.4. Mediation Analysis Controlling for Perceived Helplessness in ICU

A mediation model with the recalled experience of a traumatic event from the ICU as independent variable, diagnosis of MDD as a dependent variable and perceived helplessness during intensive care as a mediator, was tested. The recalled traumatic experience from the ICU was positively associated with the diagnosis of MDD (standardized beta = 1.03; *Z*(128) = 2.14; *p*=0.0321; 95% CI 0.09–1.98) as well as with the perceived helplessness (standardized beta = 2.63; *t*(128) = 4.06; *p* < 0.001; 95% CI 1.35–3.91). Perceived helplessness was also positively related to the outcome variable (standardized beta = 0.16; *Z* = 2.07; *p*=0.0383; 95% CI −0.3566 to 1.6761). The direct effect of the recalled traumatic experience from the ICU on the diagnosis of MDD became nonsignificant when controlling for perceived helplessness, suggesting full mediation (beta = 0.66; *Z*(128) = 1.27; *p*=0.203; 95% CI −0.36 to 1.68). However, the Sobel test failed statistical significance (*Z* = 1.84; SE = 0.22; *p*=0.065).

## 4. Discussion

The present study investigated the frequency and predictors of MDD in chronically critically ill (CCI) patients, following prolonged mechanical ventilation in ICU. Currently, there is a paucity of data facing depressive symptoms as long-term mental health consequence in these patients. MDD occurs as part of the post-intensive care syndrome (PICS) and can be related to a delayed physical recovery after intensive care [38].

Our main results reveal that every fifth CCI patient suffers from a full- or subsyndromal manifestation of MDD up to six months after transfer to post-acute ICU. Furthermore, the perceived helplessness in ICU and a diagnosis of PTSD up to six months following ICU turned out to be significant predictors of MDD in CCI patients. In a multivariable model, perceived helplessness and a diagnosis of PTSD led, respectively, to a two- and seven-fold increase in the risk to develop a MDD.

The present results suggest a rate of 17.6% of a full- or subsyndromal manifestation of MDD in CCI patients in the long-term course. This rate falls within the range of point prevalence for clinically significant depressive symptoms found in survivors of the acute respiratory distress syndrome (ARDS) (systematic review [7]: 17% to 43%; [35]: 16%]. Our rate is lower than the median point prevalence of 28% and 30%, respectively, reported for general ICU survivors (for systematic reviews, see [14, 20]). Most of the considered research used self-report instruments in order to ascertain the diagnosis of MDD. Studies using the SCID I, as in our study, showed a much lower point prevalence (e.g., 4% [30]). Additionally, these diverging rates could be partly ascribed to different follow-up periods. Former studies used a longer follow-up period (e.g., median follow-up of eight years) than our quite short period of six months after ICU [30]. In line with this, research also showed that depressive symptoms decline from the time of the acute medical illness [39]. It might be assumed that during the first six months after being discharged from ICU, patients experience a tremendous unpredictability regarding relapses, as well as the future financial and family support. Thus, depressive symptoms can be launched. Moreover, patients are confronted with a possible loss of the ability to work, transitions to special care homes, the need for intensive outpatient care, and physical or occupational therapy. In the absence of adaptive coping strategies and socioemotional resources to face the new demands, depressive symptoms might occur and may lead to additional psychosocial problems, decreasing the patients' quality of life [40, 41]. In the present study, a negative association between MDD and the health-related quality of life was revealed for CCI patients and confirms former results in survivors of the ARDS [7].

The main finding of the present study is that an acute stress reaction during the ICU stay, comprising symptoms of acute stress disorder, and perceived helplessness, significantly predicts the development of MDD up to six months after ICU. To our knowledge, this is the first study showing that a clinical diagnosis of acute stress disorder is a risk factor for MDD as mental long-term sequela in CCI patients. Acute psychological reactions such as extreme fear in the ICU and the impact of the mood in ICU (e.g., anxiety, depression, and confusion) have already been pointed out to be the strongest risk factors for developing mental illness in mixed-diagnosis patients after discharge from general ICU ([17]; for a systematic review and meta-analysis, see [20]). However, it is not clear which kind of stress reaction mediates the association between the traumatic experiences from the ICU and the subsequent psychiatric morbidity. In our own study with CCI patients [10] and in a study by Samuelson et al. [19] on mechanically ventilated adults, extreme fear of dying and signs of agitation at ICU were proven as significant acute psychological risk factors for PTSD symptoms. With respect to depressive symptoms, perceived helplessness as acute stress reaction in ICU, seems to be the main determinant, mediated for the impact of the traumatic experience in ICU. CCI patients are often unable to express what they desire because of communication disabilities. This finding is in accordance with the theory of learned helplessness, postulated by Abramson et al. [42]. In this model of depression, experiences of uncontrollability, or response-outcome independence, are assumed to lead to depressive symptoms like passivity, or depressed effect. An acute medical illness and the treatment in ICU are major stressful events associated with a profound loss of control, as well as the experience of universal or personal helplessness. In combination with medication, proinflammatory processes, encephalopathy or hypoxemia, traumatic experiences, and their interpretation may lead to long-term psychiatric morbidity following ICU [43]. The acute stress disorder and peri-ICU mood may be regarded as early markers and manifestations of future psychopathology [17].

Another important finding of the present study is that every second CCI patient with MDD exhibits comorbid PTSD. This is in line with previous studies (e.g., Shalev et al. [44]), showing a comorbidity rate of 43% in general trauma survivors. Accordingly, post-ICU PTSD symptoms were also strongly correlated cross-sectionally with post-ICU depressive symptoms [19, 38]. It is further known that depression and PTSD can frequently co-occur following a traumatic event [45], and MDD is the most frequent concurring psychiatric condition in patients with PTSD [46].

Neither age, gender, and educational level nor the severity of the medical illness emerged as clinically relevant risk factors for MDD. This is in line with existing findings in ARDS patients, medical/surgical/trauma patients, or general ICU survivors [7, 14, 17, 35, 47]. However, former studies also proved younger age, female gender, and a lower socioeconomic status as being significantly related to clinically relevant symptoms of depression (e.g., [17, 35]). Differences between findings may be ascribed to methodical inconsistencies; for example, socioeconomic position was assessed using the National Statistics Socio-economic Classification in a former study [17], whereas in our present study, only indirect assessment via the educational level was realized.

Moreover, neither a longer duration of mechanical ventilation nor the length of ICU stay could be proven as significant risk factors for MDD in our sample of CCI patients. This result contradicts findings showing an association between depressive symptoms and these clinical variables [7, 35, 39, 48]. One reason for these inconsistent findings might be differences in the study design and study samples. Nelson et al. [48] and Weinert et al. [39] investigated patients following an acute lung injury using a cross-sectional design, with retrospective medical record abstraction. Furthermore, mainly self-report measures, instead of a structured clinician-based interview, were used for the assessment of MDD [7, 35]. It should also be taken into consideration that clinical variables such as the duration of mechanical ventilation were rather associated with symptoms of anxiety, than depression following ICU treatment [35].

We did not find a significant association between prior psychiatric history and the evolvement of MDD within up to six months following ICU visit, as shown in several existing studies [15, 17, 35]. However, Jackson et al. [16] demonstrated that depression is common even among those ICU patients with no proxy reported history of depression. One reason for these diverse findings might be ascribed to the method of assessment. Most studies relied on patient's health records, instead of the assessment of the lifetime mental morbidity, with a clinician-administered semistructured interview, as done in the present study. Also, sepsis did not display a significant risk factor for MDD in CCI patients. This confirms former results showing that neither incident sepsis nor sepsis-related clinical characteristics were significantly associated with depressive symptoms in survivors of severe sepsis [15].

Our results have to be crucially evaluated in the context of methodical strengths and shortcomings. One major strength of the present study is the assessment of the diagnosis of MDD by means of the SCID I, instead of a symptom questionnaire only. Our study was longitudinally designed, current psychopathology was prospectively assessed up to the suggested interval of six months [40], and a quite homogeneous sample of CCI patients was investigated.

The following methodical limitations should be considered: despite the fact that patients were assessed for MDD at three and six months, after being transferred from acute ICU to post-acute ICU, they were not separately considered in statistical analyses in order to increase the number of cases. This may have led to an overestimation of MDD. Future studies should therefore increase the sample size in order to precisely evaluate the natural history of post-ICU psychopathology, at more than one time period [14]. Additionally, perceived helplessness in ICU as well as the lifetime history of depression, alcohol consumption, and anxiety was assessed retrospectively. Future research should prospectively measure pre-CCI psychopathology in order to prevent memory bias. Likewise, future studies should additionally use a self-rating questionnaire in order to more profoundly evaluate cognitive and somatic symptoms of depression (e.g., Beck Depression Inventory II [16]) and thus, corroborate post-ICU depressive symptoms as risk factors for post-ICU depression in CCI patients [14]. Furthermore, the regime of peri-ICU medication (e.g., use of benzodiazepines, hydrocortisone, vasopressors, and opioids) should be rigorously determined in future studies with CCI patients [17]. Additionally, generalizability of the present results is limited by peculiarities of the patient sample. Nearly three quarters of the CCI patients consist of men who are possibly less willing to report depressive symptoms, leading to lower MDD rates [49]. Finally, the present results have to be cautiously evaluated since patients who dropped out had longer ICU stays, lower education, lower Barthel index, and more often chronic kidney disease, which may have led to an underestimation of depression.

## 5. Conclusions

Our results reveal that nearly every fifth chronically critically ill patient is affected by a MDD up to six months following long-term mechanical ventilation in ICU. Consequently, MDD should be regularly evaluated during the first six months after ICU in these patients. This is of major clinical relevance since MDD as part of the PICS can negatively affect patients' adherence and motivation to appropriately cooperate in the rehabilitation process, thus leading to an adverse medical outcome and further increasing the mortality risk [50]. Early psychological risk markers for MDD such as symptoms of acute stress disorder, symptoms of PTSD, or perceived helplessness in ICU should be assessed at an early stage after leaving acute ICU in CCI patients. Future studies should validate screening tools assessing these risk markers. Furthermore, supportive interventions increasing the sense of control in ICU patients or improving their communicative skills during the ICU stay should be developed and evaluated.

## Figures and Tables

**Table 1:** Sociodemographic and clinical characteristics of the patients being followed up six months after the transfer from acute ICU to post-acute ICU (*N*=131) and dropouts (*n*=81).

Characteristics	Patients followed up (*n*=131)	Dropouts (*n*=81)	*U*/*χ* ^2^	*p*
Age (yrs), median (IQR)	61.2 (55.4–64.9)	61.8 (57.0–66.5)	4749.000	0.200 (*U*)^a^
Gender, *n* (%)				
Male	94 (71.8)	58 (71.6)		
Female	37 (28.2)	23 (28.4)	0.001	0.981 (*χ* ^2^)^b^

Family status, *n* (%)				
Single	12 (9.2)	10 (12.3)		
Married/cohabited	94 (71.1)	55 (67.9)		
Divorced/living apart	17 (13.0)	12 (14.8)		
Widowed	8 (6.1)	4 (4.9)	0.910	0.923 (*χ* ^2^)^b^

Education, *n* (%)^c^				
<10 yrs	39 (31.5)	39 (50.6)		
≥10 yrs	85 (68.5)	38 (49.4)	7.372	**0.007** ^*∗∗*^ (*χ* ^2^)^b^

ICU stay (days), median (IQR)	62.0 (47.0–90.0)	74.0 (54.0–114.5)	4416.000	**0.040** ^*∗*^ (*U*)^a^

Mechanical ventilation (days), median (IQR)	45.0 (28.0–70.0)	51.0 (33.0–83.0)	4639.000	0.125 (*U*)^a^

Sepsis, *n* (%)				
No sepsis	44 (33.6)	23 (28.4)		
Sepsis	48 (36.6)	32 (39.5)		
Severe sepsis or septic shock	39 (29.7)	26 (32.1)	3.270	0.352 (*χ* ^2^)^b^

Site of infection, *n* (%)				
Respiratory	63 (48.1)	48 (59.3)	2.502	0.114 (*χ* ^2^)^b^
Urinary/genitals	13 (9.9)	6 (7.4)	0.388	0.533 (*χ* ^2^)^b^
Abdominal	12 (9.2)	6 (7.4)	0.198	0.656 (*χ* ^2^)^b^
Bones/soft tissue	7 (5.3)	3 (3.7)	0.299	0.745 (†)^d^
Wound infection	2 (1.5)	1 (1.2)	0.031	1.000 (*χ* ^2^)^d^
Heart	1 (0.8)	2 (2.5)	1.044	0.559 (*χ* ^2^)^d^
Multiple	12 (9.2)	11 (13.6)	1.011	0.315 (*χ* ^2^)^b^
Others^e^	9 (6.9)	9 (11.1)	1.159	0.282 (*χ* ^2^)^b^
Unknown	4 (3.1)	3 (3.7)	0.066	1.000 (*χ* ^2^)^b^

Barthel index, median (IQR)				
At admission at post-acute ICU, median (IQR)	−175.0 (−225.0 to −95.0)	−200.0 (−225.0 to −127.5)	4850.500	0.289 (*U*)^a^

At discharge from post-acute ICU at rehabilitation hospital, mean (IQR)	−30.0 (−80 to 15.0)	−55.0 (−120.0 to −10.0)	4217.500	**0.012** ^*∗*^ (*U*)^a^

At discharge from rehabilitation hospital, median (IQR)	65.0 (25.0 to 85.0)	20.0 (−55.0 to 65.0)	3083.500	**<0.001** ^*∗∗∗*^ (*U*)^a^

^a^ *p* value from the Mann–Whitney *U* test; ^b^ *p* value from *χ* ^2^-test; ^c^patients followed up: *n*=7 missing values; patients dropped out: *n*=4 missing values; ^d^ *p* value from Fisher's exact test; ^e^patients followed up: brain (*n*=1), central venous catheter (*n*=7), and portsystem (*n*=1); patients dropped out: central venous catheter (*n*=5), intracardiac catheter (*n*=1), nose (*n*=1), portsystem (*n*=1), and aorta (*n*=1); IQR = interquartile range; ASDS = Acute Stress Disorder Scale; ^*∗*^ *p* ≤ 0.05; ^*∗∗*^ *p* ≤ 0.01; ^*∗∗∗*^ *p* ≤ 0.001.

**Table 2:** Frequency of major depressive disorder (MDD) at *t*2 (three months after the transfer from acute ICU) and at *t*3 (six months after the transfer from acute ICU) and summarized at *t*2 or *t*3.

	*t*2	*t*3	*t*2 or *t*3
Number of CCI patients interviewed	*n*=123	*n*=109	*n*=131
Number of patients with full-syndromal MDD (%)	*n*=6 (4.9%)	*n*=6 (5.5%)	*n*=10 (7.6%)
Number of patients with subsyndromal MDD (%)	*n*=6 (4.9%)	*n*=7 (6.4%)	*n*=13 (9.9%)
Number of patients without MDD (%)	*n*=111 (90.2%)	*n*=96 (88.1%)	*n*=108 (82.4%)

**Table 3:** Multivariable logistic regression using the forward Wald method for the identification of predictors of a major depressive disorder (MDD) in chronically critically ill (CCI) patients three to six months after the transfer from acute ICU to post-acute ICU.

Multivariable logistic regression			
	OR	CI	*p* value
*Psychological variables at (post-acute) ICU*			
Perceived helplessness in ICU^1^	1.792	1.08–2.971	**0.024^∗^**
*Psychological variables three to six months after discharge from post-acute ICU*			
Diagnosis of PTSD according to the SCID I	7.414	2.63–20.94	**<.001^∗∗∗^**
Cox and Snell *R* ^2^	0.153		
Nagelkerke's *R* ^2^	0.252		
−2 log-likelihood	99.458		

^1^Method of multivariable logistic regression analysis: Wald forward; SCID I = Structured Clinical Interview for DSM-IV; ^*∗*^ *p* ≤ 0.05; ^*∗∗∗*^ *p* ≤ 0.001.

## Abbreviations

ARDS: Acute respiratory distress syndrome
ASDS: Acute Stress Disorder Scale
CAM-ICU: Confusion Assessment Method for the Intensive Care unit
CCI: Chronic critical illness
CIM: Critical illness myopathy
CIP: Critical illness polyneuropathy
DSM-IV: Diagnostic and Statistical Manual of Mental Disorders-IV
EQ-5D-3L: Euro-Quality of Life
ICU: Intensive Care Unit
MDD: Major depressive disorder
MSPSS: Multidimensional Scale of Perceived Social Support
PICS: Post-intensive care syndrome
PTSD: Posttraumatic stress disorder
SCID I: Structured Clinical Interview for DSM-IV.

## Supplementary Materials

Supplementary Materials Table S1: medical comorbidities of the patients being followed up (*n*=131) and the dropouts (*n*=81). Table S2: univariate logistic regression using Enter as a method for the identification of sociodemographic, clinical, and psychological predictors of a major depressive disorder (MDD) in chronically critically ill (CCI) patients three to six months after the transfer from acute ICU to post-acute ICU.
