# Supplementary material for: Predictors of Major Depressive Disorder following Intensive Care of Chronically Critically Ill Patients
Source: Crit Care Res Pract. 2018 Aug 1;2018:1586736. doi: 10.1155/2018/1586736 (PMC6093074; doi:10.1155/2018/1586736)
Supplement: Supplementary Materials — Table S1: medical comorbidities of the patients being followed up (n=131) and the dropouts (n=81). Table S2: univariate logistic regression using Enter as a method for the identification of sociodemographic, clinical, and psychological predictors of a major depressive disorder (MDD) in chronically critically ill (CCI) patients three to six months after the transfer from acute ICU to post-acute ICU. [file 1586736.f1.doc]

**Supplementary material**

**Table S1:** Medical comorbidities of the patients being followed-up (n = 131) and the drop outs (n = 81).

**Table S2:** Univariate logistic regression using Enter as method for the identification of sociodemographic, clinical and psychological predictors of a Major Depressive Disorder (MDD) in Chronically Critically Ill (CCI) patients three to six months after the transfer from acute care ICU to post-acute ICU.

**Table S1:** Medical comorbidities of the patients being followed-up (n = 131) and the drop outs (n = 81).

| **Characteristic** | **Patients followed-up**  **n = 131** | **Drop Outs**  **n = 81** | **χ²** | ***P*** |
| --- | --- | --- | --- | --- |
| **Medical comorbidiy** |  |  |  |  |
| **Lung** |  |  |  |  |
| Chronic obstructive pulmonary disease (COPD) (J44.X) | 39 (29.8) | 30 (37.0) | 1.204 | .273 (χ²)^a^ |
| Acute respiratory insufficiency (J96.00, J96.01, J96.09) | 101 (77.1) | 65 (80.2) | .292 | .589 (χ²)^a^ |
| Chronic respiratory insufficiency (J96.10,J96.11, J96.19) | 11 (8.4) | 7 (8.6) | .004 | .950 (χ²)^a^ |
| Pneumonia (J15,J18,J69) | 23 (17.6) | 24 (29.6) | 4.228 | **.040*** (χ²)^a^ |
| Sleep apnea (G47.3) | 16 (12.2) | 5 (6.2) | 2.047 | .153 (χ²)^a^ |
| **Diseases of the circulatory/ cardiovascular system** |  |  |  |  |
| Left heart failure (I50.1) | 43 (32.8) | 28 (34.6) | .068 | .794 (χ²)^a^ |
| Atrial fibrillation (I48.0-I48.2) | 43 (32.8) | 27 (33.3) | .006 | .939 (χ²)^a^ |
| Hypertension (I10.0, I10.01) | 79 (60.3) | 40 (49.4) | 2.425 | .119 (χ²)^a^ |
| Coronary heart disease (I25.1)^1^ | 37 (28.2) | 31 (38.3) | 2.310 | .129 (χ²)^a^ |
| **Kidney** |  |  |  |  |
| Chronic kidney disease (N18.X) | 31 (23.7) | 32 (39.5) | 6.014 | **.014*** (χ²)^a^ |
| Urinary tract infection (N39.0) | 27 (20.6) | 26 (32.1) | 3.523 | .061 (χ²)^a^ |
| **Other** |  |  |  |  |
| Diabetes (E11.90) | 45 (34.4) | 23 (28.4) | .815 | .367 (χ²)^a^ |
| Adipositas (E66) | 32 (24.4) | 17 (21.0) | .333 | .564 (χ²)^a^ |
| Enzephalopathy (G93.4) | 28 (21.4) | 23 (28.4) | 1.351 | .245 (χ²)^a^ |
| Brain damage | 49 (37.4) | 40 (49.4) | 2.948 | .086 (χ²)^a^ |
| Neurological disorders | 31 (23.7) | 30 (37.0) | 4.368 | **.037*** (χ²)^a^ |
| cirrhosis of the liver | 2 (1.5) | 4 (4.9) | 2.118 | .205 (†)^b^ |
| **Prior psychiatric history** |  |  |  |  |
| History of Major Depressive Disorder | 30 (22.9) | 21 (25.9) | .251 | .617 (χ²)^a^ |
| History of Anxiety Disorder | 11 (8.4) | 9 (11.1) | .432 | .511 (χ²)^a^ |
| History of harmful alcohol consumption | 26 (19.8) | 17 (21.0) | .040 | .841 (χ²)^a^ |

^a^*p*-value from χ²-test; ^b^*p*-value from Fisher´s exact test; ^, *p ≤ .05^

**Table S2:** Univariate logistic regression using Enter as method for the identification of sociodemographic, clinical and psychological predictors of a Major Depressive Disorder (MDD) in Chronically Critically Ill (CCI) patients three to six months after the transfer from acute care ICU to post-acute ICU.

| **Univariate logistic regression** | | | |
| --- | --- | --- | --- |
|  | **OR** | **CI** | **P value** |
| **Sociodemographic variables** |  |  |  |
| Age | .93 | .60-1.44 | .729 |
| Gender | 1.45 | .56-3.79 | .445 |
| Family status, no partnership vs. partnership | 3.06 | .85-11.01 | .086 |
| Education status < 10 yrs vs. ≥ 10 yrs^1^ | 1.09 | .38-3.07 | .879 |
| **Clinical variables** |  |  |  |
| sepsis, yes vs. no | .94 | .36-2.42 | .894 |
| Kind of sepsis |  |  |  |
| Sepsis | 1.76 | .70-4.37 | .224 |
| Septic shock | .84 | .17-4.07 | .828 |
| Severe sepsis | .33 | .07-1.52 | .157 |
| Number of sepsis episodes | .89 | .53-1.48 | .646 |
| Barthel index at admission at post-acute ICU | .68 | .41-1.13 | *.*137 |
| Barthel index at discharge from post-acute ICU | .82 | .55-1.23 | .345 |
| Length of mechanical ventilation | .84 | .51-1.39 | .503 |
| Length of ICU stay | .74 | .44-1.25 | .265 |
| **Psychological variables in (post-acute) ICU** |  |  |  |
| CAM-ICU sum score | .42 | .06-2.85 | .375 |
| perceived helplessness in ICU^2^ | 1.88 | 1.17 -3.02 | **.009**** |
| perceived fear of dying in ICU^3^ | 1.32 | .86-2.02 | .209 |
| ASDS > 56, yes vs. no | 2.43 | .42-14.13 | .323 |
| Symptoms of Acute Stress Disorder according to the ASDS^3^ | 1.62 | 1.10-2.37 | **.015*** |
| Diagnosis of ASD according to SCID I^3^ | 3.05 | .99-9.35 | .051 |
| Recalled experience of a traumatic event from the ICU | 2.69 | 1.05-6.90 | **.039*** |
| **Psychological variables three to six months after discharge from post-acute ICU** |  |  |  |
| Number of traumatic memories^3^ | 1.43 | .90-2.28 | .130 |
| Perceived social support according to MSPSS^3^ | .99 | .63-1.56 | .980 |
| Diagnosis of PTSD according to SCID I | 7.33 | 2.72-19.76 | **<.001***** |
| **Prior psychiatric history** |  |  |  |
| History of depressive disorder | .92 | .31-2.74 | .884 |
| History of harmful alcohol consumption | .82 | .25-2.67 | .745 |
| History of anxiety disorder | 1.05 | .21-5.20 | .955 |

Method of univariate logistic regression analysis: Enter; 1 n = 7 missing values; 2 n = 1 missing value; 3 n = 2 missing values;

ASDS = Acute Stress Disorder Scale; ASD = Acute Stress Disorder; MSPSS = Multidimensional Scale of Perceived Social Support Multidimensional; SCID I = Structured Clinical Interview according to DSM IV; Diagnosis of ASD according to SCID I was highly significantly correlated with ASDS score (point-biserial correlation r = .774, p < .001). For parsimony of the final model, only the variable ASDS score was considered., *p ≤ .05, **p≤ .01, ***p ≤ .001
